# Prevalence of Otitis Externa in a Population of Owned Cats in Northern Italy

**DOI:** 10.3390/ani16050706

**Published:** 2026-02-24

**Authors:** Roberta Perego, Eva Spada, Claudia Avizzano, Luciana Baggiani, Daniela Proverbio

**Affiliations:** Department of Veterinary Medicine and Animal Sciences (DIVAS), University of Milan, Via dell’Università 6, 26900 Lodi, Italy; eva.spada@unimi.it (E.S.); luciana.baggiani@unimi.it (L.B.); daniela.proverbio@unimi.it (D.P.)

**Keywords:** otitis externa, cat, prevalence, cytology, owned cat

## Abstract

Although increasingly studied feline otitis externa (OE) prevalence is not yet well defined. The aim of this study was to assess the prevalence of OE in a population of privately owned cats in Northern Italy. Cats were clinically, otoscopically and cytologically evaluated. Diagnosis of OE was based on clinical findings along with abnormal ear cytology. The influence of the main demographic variables was examined. The prevalence of OE was 17% with a positive correlation to being European, and having short hair, a dermatological history, pruritus and multiple clinical signs. Ear cytology was abnormal in 19% of cats. Mites were found in 6% of the cats, about half of which were exclusively indoor cats and completely asymptomatic. Pathological numbers of bacteria and/or yeasts were found on cytology in 15% of cats and, of these, 29% had only *Malassezia* spp., 65% only cocci, and 6% a mixed population of these microorganisms. The prevalence of OE in owned cats in Northern Italy is relatively high. The identification of parasitic OE in asymptomatic and exclusively indoor cats, and a correlation between OE and dermatological history highlights the importance of systematic ear evaluation as part of routine feline health assessments.

## Simple Summary

Feline otitis externa (OE) is a dermatological disease that has not been fully defined. The aim of this study was to assess the prevalence of OE in a population of privately owned cats presented to a teaching hospital in Northern Italy. Diagnosis of OE was based on clinical findings along with abnormal ear cytology and the influence of the main demographic variables was evaluated. Two hundred and four cats were recruited and examined clinically, otoscopically and cytologically. The prevalence of OE was 17% with a positive correlation to being European and having short hair, a history of dermatological problems, pruritus and multiple clinical signs. Ear cytology was abnormal in 19% of cats. Ear mites were found in 6% of cats, about half of which were exclusively indoor and asymptomatic. Pathological numbers of bacteria and/or yeasts were found in 15% of cats. OE prevalence in owned cats in Northern Italy is relatively high, but significantly lower than in the stray cats in the same geographical area. The identification of parasitic OE in asymptomatic and exclusively indoor cats, and of a correlation between OE and dermatological history, highlights the importance of systematic ear evaluation as part of routine feline health assessments.

## 1. Introduction

Otitis externa (OE) is an inflammation of the auricular pinna and the external ear canal with a multifactorial etiopathogenesis characterized by primary, secondary and perpetuating factors. It represents a significant clinical problem in small animal practice [1]. Primary causes of OE include congenital anatomical alterations, hypersensitivity disorders, foreign bodies, mites and less frequent systemic or immune-mediated conditions [1,2].

*Otodectes cynotis* is the main primary agent responsible for feline OE [3], with a very variable geographical prevalence, reportedly ranging from 0.9% in England/Wales and Australia [4,5], to 2% in Belgium [6], 2.2% in Portugal [7], 13.9% in Brazil [8], 25.7% in Croatia [9], 29.4% in Italy [10], 37% in Florida [11] and 83.7% in UK [12]. Many of these studies, however, are on feral or stray cat populations [5,6,9,10], which may not be representative of the owned feline population seen in primary veterinary care or referral practice.

*Malassezia*species are known to be part of the normal aural microflora in cats and an important causative agent of OE [13,14,15,16]. *Staphylococcus pseudintermedius*, *Proteus mirabilis*, *Pseudomonas aeruginosa*, *Corynebacterium* spp. and *Escherichia coli* are the most commonly reported secondary agents in feline OE [7,17,18]. Many studies have used bacterial and fungal cultures to provide epidemiological investigation of the aural microflora of cats, with and without OE [14,19,20,21,22,23], but fewer studies [4,6,10,24] have examined ear cytology from different feline populations, reporting prevalences from 37.8% in rescue/owned cats [4] to 74% in stray cats [6] for *Malassezia*species and from 2.6% [4] to 9% [6] for bacteria, respectively, in rescue/owned cats and in stray cats.

Clinical signs of feline OE usually include intense auricular pruritus, with ear scratching and the possible presence of self-induced injuries and head shaking [6,7]. The diagnosis of feline OE is established by direct otoscopy, that allows for the observation of a sticky, strong-smelling exudate with or without canal wall hyperemia and hyperplasia, stenosis or masses in the ear canal [1], and by microscopic evaluation of ear secretions [25], which reveals abnormal cytological findings [15,16]. However, many cats with OE are presented to a veterinary practitioner for reasons unrelated to the ear canal [4], otoscopy does not always reveal significant changes in the course of feline otoacariasis [4,11], the presence of cerumen is not always related to OE [4] and the cytological counts of bacteria and yeast that differentiate a feline ear affected by otitis from a healthy one derive from dated studies on a very small number of cats [15,16].

Epidemiologically speaking, OE in cats has traditionally been considered less common than in dogs [26,27,28,29]; however, growing evidence suggests that the prevalence of OE in cats may be underestimated, particularly in owned cat populations [2,30]. Studies on the prevalence of OE in cats are limited, often outdated and methodologically heterogeneous, with insufficient characterization of study populations and diagnostic criteria [31]. A few older studies report the prevalence of feline OE to be between 2% and 10% [26,28]. More recent investigations have reported a wide range of prevalence values, from 25.5% in privately owned cats in Brazil [8] to 55.1% in Italian stray cats [10].

Geographical variability is also likely to influence the prevalence and presentation of OE in cats. Climate, humidity, population density, and access to routine veterinary care may affect both the frequency of the disease, and the distribution of underlying causes [30,31].

In Europe, and particularly in Italy, there is a large population of owned cats; nevertheless, contemporary population-based epidemiological data on feline OE are scarce [10,13]. Northern Italy, characterized by a temperate climate with seasonal humidity [32] and the presence of indoor–outdoor cats [33], provides a relevant setting for investigating this condition.

The objective of this study was to assess the prevalence of OE in a population of owned cats in Northern Italy based on ear cytology and clinical findings, as well as to identify the main parasitic/infectious factors related to the development of OE. The analysis considered potentially significant anamnestic and signalment factors, and the results were compared with a previous study on stray cats in the same geographical area. By providing updated epidemiological data from a defined geographic area, this study aimed to contribute to a better understanding of feline OE and to support improved preventative, diagnostic, and therapeutic strategies in clinical practice.

## 2. Materials and Methods

Privately owned cats of both sexes of different breeds and ages examined at the Veterinary Teaching Hospital of the University of Milan between January 2023 and December 2024, regardless of history, presenting complaint and the presence/absence of owner-reported symptoms and clinical signs of otitis externa, were included. All samples were collected as a routine part of the feline clinical examination, with oral informed consent obtained from the cats’ owners. Thus, for this study, authorization from the University Animal Welfare Organization (OPBA) was not required. Stray cats, rescue cats or owned cats with an uncooperative nature, on which, therefore, a complete clinical and otoscopic evaluation was not possible, were excluded from the study. For each cat included in the study the presenting complaint, the past and recent medical history (for identification of previous dermatological problems) and owner-reported symptoms of OE (Table 1) were recorded. Additional information collected included: sex (male/female), neutering status (neutered/intact), age and age group (kitten [0–1 years], young adult [1–6 years], mature adult [7–10 years], and senior [>10 years]) [34], breed, hair length (short/medium/long hair), lifestyle (indoor or outdoor) and sampling season (spring/summer/autumn/winter).

Clinical examination was performed to establish general health (healthy/unhealthy) and to identify and record clinical signs of OE (Table 1). Otoscopic assessment (Heine G100 LED, Gilching, Germany) of the external ear canal was used to identify normal/altered ears. In detail, during the otoscopic assessment, the ear canal alterations and the amount of cerumen, graded according to the literature (Table 1), were recorded. If it was not possible to evaluate the tympanic membranes due to the presence of excess cerumen or ear canal masses that information was recorded.

Ear material was collected using non-sterile swabs inserted into the lumen of the ear canal and swabbed against the surface of the vertical canal. The cotton swab was rolled onto two clean microscope slides, evenly distributing a thin layer of material. One unstained sample was suspended with mineral oil and mounted with a cover slip for examination for mites and the other slide was stained with a modified Wright’s rapid stain (Quick Panoptic kit; Pokler, Salerno, Italia), suspended with mineral oil, and mounted with a cover slip for morphological identification of microorganisms such as yeasts of the *Malassezia* genus, cocci or bacilli, and inflammatory cells. Both slides were immediately examined.

The unstained slide was scanned at low power (100×) for qualitative parasitological evaluation (positive or negative), i.e., the identification of a single mite (*O. cynotis*), adult, or immature (eggs, larvae, and nymphs) was considered positive for infection.

Stained slides were evaluated first at low power (100×) to identify the areas of interest and then under high-power (400×) to quantify morphologically identifiable microorganisms such as *Malassezia*species and/or bacteria and inflammatory cells. Ten fields were examined. For each slide the total number of microorganisms was recorded (the sum of all 10 fields) and the mean number per high power field (HPF) was calculated. Cytology was considered pathological using the following limits, as they remain the only published semiquantitative criteria for feline ear cytology [15]: *Malassezia* spp. ≥12/HPF, cocci ≥15/HPF, and rods ≥1/HPF. When microorganisms exceeded 100 units/HPF, the approximate value of 100 microorganisms/HPF was entered into the database. A high-power oil immersion field (1000×) was used only for accurate identification of the kind of bacteria (cocci or rods). Inflammatory cells were noted as being present or absent for the whole of the slide. Assessments were performed on both ears (right and left) of each cat and data were separately collected and tabulated to differentiate results by ear.

A cat was classified as having OE if at least one ear met one of the following criteria: (1) presence of at least one owner-reported symptom and/or clinical sign of OE (Table 1) along with a pathological cytology for yeast and/or bacteria (*Malassezia* spp. ≥12/HPF, cocci ≥15/HPF, rods ≥1/HPF); (2) presence of at least one mite (adult of immature form) or inflammatory cells with or without clinical symptoms/signs. Cats that did not meet one of these criteria were not considered to have OE.

Assessments, collection, slide preparation, and microscopic observations were performed by the same evaluator.

The prevalence of OE was calculated. Although both ears were examined and recorded separately, all inferential statistical analyses were conducted at the cat level. A cat was considered positive for OE if at least one ear met the diagnostic criteria. Ear-level data were used only for descriptive cytological summaries. The data were analyzed descriptively, and associations between OE, epidemiological variables selected for this study such as age group, sex, reproductive status, breed, lifestyle, sampling season and microorganism involved (*Malassezia*species/cocci/rods/mites species) and clinical symptoms/signs were assessed using univariate chi-square or Fisher’s exact tests, with *p* set at 0.05. Given the exploratory nature of the study and the limited number of OE cases, multivariable logistic regression was not performed. The authors acknowledge the increased risk of a type I error due to multiple univariate comparisons; therefore, results should be interpreted as exploratory. To identify possible risks and protective factors associated with OE, a univariate analysis was conducted and odds ratios (OR) with 95% confidence intervals (95% CI) were calculated for each variable statistically significantly linked to the otitis status. The concordance between the presenting complaint, clinical symptoms/signs, and abnormal cytological findings was binary-analyzed using Cohen’s Kappa coefficient with 95% CIs. The mean (±SD) number of yeasts or bacteria per ear (right and left) and subsequently for both ears combined was calculated, in cats with otitis and cats without otitis, tested for normal distribution with the d’Agostino-Pearson test and evaluated with an independent samples *t*-test. All data were analyzed using commercial software (MedCalc Software v.11.5.1, Mariakerke, Belgium).

## 3. Results

Two-hundred and four cats with a mean age of 5.3 ± 4.8 years, ranging from 2 months to 18 years, were examined in this study and their epidemiological data are reported in Table 2.

The prevalence of OE was 17% (35/204 cats; 95% CI: 12.1–22.7), with a statistically positive correlation with being European (*p* = 0.03) and having short hair (*p* = 0.03) with an OR of 3.8 and 6.8, respectively. Also, having a history of dermatological problems was statistically correlated with OE (*p* = 0.0008, OR = 4.6), although only 5% (10/192) of cats had suspected OE as the presenting complaint. In contrast, the spring season appeared to be negatively correlated with the development of OE (*p* = 0.01). Among the 35 cats diagnosed with OE, the diagnostic criteria were met as follows: 23/35 (66%) based on clinical symptoms and/or signs along with pathological cytology for bacteria and/or yeasts and 12/35 (34%) based on the presence of mites (alone or associated with a pathological number of bacteria or yeast).

Owner-reported symptoms or clinical signs consistent with OE at the time of examination were seen in 22% (44/204) of cats, with a strong statistically positive correlation between the final diagnosis of OE and the presence of ear discharge (*p* = 0.0000, OR: 48), pruritus (*p* = 0.0000, OR: 39), and abundant cerumen (*p* = 0.0000, OR: 56), as well as erythema (*p* = 0.0000, OR: 12), stenosis (*p* = 0.0000, OR: 76) and ulcerations (*p* = 0.04, OR: 13) of the ear canal and pinna crust/erosions (*p* = 0.0065, OR: 19.6). On the other hand, a statistically significant negative association was observed between OE and a small amount of cerumen (*p* = 0.0002), no ear symptoms (*p* = 0.0000) and no ear alterations (*p* < 0.0001) (Table 3).

The tympanic membrane was not visible in 7% (15/204) of cats and no masses were found in any of the cats evaluated during direct otoscopy.

No microorganisms were found in either ear of 83/204 (41%) cats and in 83/204 (41%) cats bacteria or yeast were found in at least one ear but not in pathological numbers, whereas ear cytology was abnormal (simple presence of at least one mite and/or bacteria/*Malassezia* in pathological numbers) in 19% (38/204) of cats. The presence of an abnormal ear cytology showed very good agreement with the final diagnosis of otitis (K = 0.9, CI: 0.87–0.99). In contrast, agreement between suspected OE as the presenting complaint and clinical symptoms/signs of OE showed low agreement (K = 0.3, CI: 0.13–0.46), as did suspected OE as the presenting complaint and abnormal cytology (K = 0.2, CI: 0.03–0.39). There was a good agreement between clinical symptoms/signs consistent with OE and abnormal cytology (K = 0.7, CI: 0.53–0.79).

In 6% (12/204) of cats, mites were found in the ear canal, either alone (7/12) or associated with a pathological number of bacteria or yeast (5/12). Of these 11/12 had *Otodectes cynotis* and 1/12 had *Demodex cati*; half of the cats in which mites were identified (6/12) were exclusively indoor cats and 5/12 cats were completely asymptomatic.

Fifteen percent of cats (31/204) had cytology showing bacteria and/or yeasts in pathological numbers, either unilaterally (35%, 11/31) or bilaterally (65%, 20/31), and of these, in 16% (5/31) this finding was associated with mites. Of the cats with pathological numbers of bacteria and yeasts, 29% (9/31) had only *Malassezia* spp., 65% (20/31) only cocci, and 6% (2/31) a mixed population of these microorganisms. No rods or inflammatory cells were observed in any slide. Table 4 shows the results of abnormal ear cytology comparing cats with and without OE and Table 5 reports the mean number per high-power field (HPF) of *Malassezia* spp., cocci and rods in cats with and without otitis.

## 4. Discussion

This study reports epidemiological, clinical and cytological data on OE in a population of owned cats presented for routine veterinary examination, confirming that feline OE is a relatively frequent, but still under-recognized, condition in clinical practice.

The prevalence of OE in owned cats observed in this study (17%) is similar to that (19%) of an old UK survey of the prevalence of dermatological conditions in small animals in general practice [29], although in the UK study no information about the diagnostic methods used or the size of the study population was reported. Conversely, an old and poorly described Romanian study in a large population of 4572 domestic cats [27] reported an OE prevalence of 2%, without details on inclusion criteria, diagnostic methods or microorganisms involved. A recent Brazilian study on the prevalence of feline OE in owned cats (25.5%) [8] is the only study with which we can directly compare our data. The other studies in the literature include different populations, for example mixed rescue and owned cats [4] or symptomatic cats [22], or use different diagnostic methods [22,35]. The slightly higher reported prevalence of OE in the Brazilian study [8] can be explained by the higher prevalence of mites (13.9%) in that study and by the different criteria used to consider the cats as having OE since, in that study [8], cats without clinical signs but with large numbers of microorganisms on cytology were also considered to have OE.

The prevalence of OE reported in our study is also significantly lower, as expected, than a previously published study on stray cats from the same geographic area (55%) [10]. This is probably due to the lower prevalence of otoacariasis, 6% versus 53.3%, in owned cats. Stray cats are unlikely to receive regular anti-parasitic treatments, will have more contact with other animals and often live in large communities, making them more susceptible to otoacariasis [11].

Among the evaluated demographic variables, breed was significantly associated with OE, with European cats showing a higher prevalence compared with other breeds. Data on breed predispositions for feline OE are limited and inconsistent and, unlike dogs, no clear conformational risk factors have been established in cats [25]. The overrepresentation of European cats in the general feline population in this study may partly account for this result.

Short hair length was also positively associated with OE in our study, although the wide confidence interval indicates substantial uncertainty, and this finding should be interpreted with caution. Hair length has not been identified as a risk factor for feline OE in previous studies [30], and its role remains unclear. It is possible that the overrepresentation of short-haired cats in our population may partly account for this result.

A medical history of dermatological disease was strongly associated with OE in this study. This finding is consistent with previous literature reports indicating that feline OE is frequently secondary to underlying skin disorders, including allergic dermatitis, parasitic disease and other inflammatory dermatoses [3,25]. The high odds ratio observed reinforces the importance of considering OE as part of a broader dermatological syndrome rather than as a primary isolated disease.

Age, sex, reproductive status and lifestyle (indoor versus outdoor access) were not significantly associated with OE, which is in agreement with previous studies [8,30]. However, a higher frequency of OE was observed in young cats (46%), consistent with other studies in which the prevalence was higher in kittens and/or young cats [6,8,10,22,27].

The findings that outdoor access does not increase the prevalence of OE can be easily explained by the fact that owned cats, especially those with outdoor access, are usually subject to regular application of anti-parasitic drugs, which significantly reduces the possibility of mite infestation, a major cause of OE in stray cats [10].

Interestingly, the spring season was negatively associated with the occurrence of OE. Seasonal patterns in feline OE have not been consistently reported, unlike in dogs where allergic otitis may show seasonal variation [36]. The reduced prevalence of OE observed during spring may hypothetically reflect seasonal patterns in owner anti-parasitic management practices, making the development of mite ear infections less likely in this season; however, this finding should be interpreted cautiously and confirmed by longitudinal studies, as treatment data were not collected in our study.

An important finding of this study is the low agreement between the presenting complaint and diagnosis of OE (K = 0.3), as well as between the presenting complaint and abnormal cytology (K = 0.2). Only a small proportion of cats were presented for suspected ear disease, despite a much higher prevalence of clinical signs and cytological abnormalities. This confirms previous observations that cats often show subtle or nonspecific signs of ear disease and tend to mask discomfort, leading to under-recognition by owners [8,25,30].

In contrast, classical clinical symptoms and signs such as pruritus, ear discharge, erythema or stenosis were significantly associated with a diagnosis of OE (K = 0.7). These findings are consistent with previous studies and underline the importance of thorough history collection, clinical and otoscopic examination in cats, even in the absence of owner-reported ear-related complaints [25].

Abundant cerumen was particularly strongly associated with a diagnosis of OE, whereas the absence of cerumen was predictive of the absence of disease. Excessive cerumen production and changes in cerumen characteristics have been described as a sensitive indicator of underlying ear pathology in cats and should prompt further diagnostic investigation [4,30].

Ear cytology was abnormal in 19% of cats and showed excellent agreement with the final diagnosis of OE (K = 0.9), confirming its diagnostic value. These results support current recommendations advocating routine ear cytology in feline patients, regardless of the presence or absence of owner-reported symptoms and evident clinical signs [3,25].

Mites, predominantly *Otodectes cynotis*, with a single case of *Demodex cati*, were detected in 6% of cats. This prevalence is lower than that in a previous similar Brazilian study on owned cats [8], that reported a prevalence of 13.9%, and this difference may be due to different ectoparasite prevention practices and the different climate in the two countries. Notably, half of the *O. cynotis*-affected cats were strictly indoor and several were asymptomatic, underlining that mite-associated otitis cannot be excluded based solely on lifestyle or clinical appearance, and confirming previous observations that *O. cynotis* infestation may occur in the absence of overt clinical signs [37] and may become chronic and difficult for owners to identify.

Pathological overgrowth of bacteria and/or yeast was identified in 15% of cats, and involved cocci and/or *Malassezia* spp., either unilaterally or bilaterally. Rods were not observed in any cat. These microorganisms have also been detected in cats without OE, but not in pathological numbers. Both *Malassezia* spp. and cocci were found in significantly higher numbers in cats with OE compared to healthy cats (*p* < 0.000001). The percentage of cats where *Malassezia* spp. was detected (29%) was lower than in the similar Brazilian study on owned cats [8] and the Italian study on stray cats [10], which reported values of 57.1% and 50.5%, respectively. In a Croatian study [9], *Malassezia* spp. were found in 30% of free-roaming cats with OE. The higher prevalence of *Malassezia* spp. in cats with OE in the Brazilian [8] and previous Italian and Croatian studies [9,10] was possibly related to higher *O. cynotis* parasitism. A study isolating *Malassezia* spp. from healthy and otitic cats found that in 63.8% of cats with otoacariasis, fungi were isolated [13]. In a study in France investigating owned cats, 15 healthy cats were examined and no *Malassezia* yeasts were detected, and bacteria were isolated from just a single ear [24]. In a study performed in the USA, 52 privately owned cats were examined using ear cytology; yeasts were detected in 83% and coccoid-shaped bacteria were detected in 71% of cats [16]. In this study the percentage of cats with OE in which cocci were detected cytologically is comparable to previous literature reports [8,10,16].

Finally, the cytological thresholds and the microscope field area (400× dry objective) used in this study follow the only published, although dated, semiquantitative criteria available for feline ear cytology [15]. However, our study identified some differences from the literature [15,16] in the mean number/HPF of *Malassezia* spp. (1.4/HPF) and cocci (1.3/HPF) in clinically healthy cats. Tater [16] indicated the median number of microorganisms per high-power dry field in a healthy cat was 0.2 and 0.3 for *Malassezia* spp. and coccoid-shaped bacteria, respectively, whereas for Ginel [15] the median number of microorganisms per high-power dry field in a healthy cat was 0.5 and 1.78 for *Malassezia* spp. and bacteria of unspecified shape, respectively. Our results, obtained in a larger number of cats than these two studies, highlight the need to update cytological criteria for diagnosing this condition in cats through the analysis of larger, well-characterized populations and/or advanced diagnostic methods.

## 5. Conclusions

The prevalence of OE in cats receiving veterinary hospital care in Northern Italy is relatively high, while the proportion of *O. cynotis* in this cohort of owned cats remains limited. *Malassezia* spp. and cocci were found in both cats with OE and healthy cats, but in significantly different numbers. Routine otoscopic examination and ear cytology appear essential for early detection of OE, particularly in cats with past dermatological history. These examinations may guide the need for further collateral exams and the initiation of an appropriate therapeutic approach. The findings of this study highlight the limited reliability of owner-reported signs. The identification of asymptomatic parasitic OE, including in indoor cats, reinforces the inclusion of systematic ear evaluation as part of all routine feline health assessments.

## Figures and Tables

**Table 1:** Symptoms and signs of otitis externa (OE) [1] and cerumen scoring system [4].

Owner-Reported Symptoms of OE	Clinical Signs of OE	Amount of Cerumen and Score
Pruritus	Ear discharge	Absent: score 1
Odor	Pinna crust and/or erosion	Small: score 2
Ear discharge	Canal erythema	Moderate: score 3
	Canal stenosis	Abundant: score 4
	Canal ulceration	

**Table 2:** Descriptive statistics of categorical variables relative to total population and to cats with or without otitis. Significant *p*-values (<0.05) in bold. It should be noted that data were not available for all variables in all 204 cats and missed data was random. The denominator used for each percentage was the number of subjects for which the data was available.

Variable n = Number of Subjects for Which Data Were Available	Categories	Total N (%) of Cats	N (%) Cats with Otitis	N (%) Cats Without Otitis	X^2^ *p* Value and OR (95% CI)
**Sex** **n = 192/204**	Female	90 (47)	19 (10)	71 (37)	*p* = 0.08
	Male	102 (53)	12 (6)	90 (47)	
**Reproductive status** **n = 192/204**	Neutered	133 (69)	22 (11)	111 (58)	*p* = 0.8
	Intact	59 (31)	9 (5)	50 (26)	
**Age group** **n = 190/204**	Kitten	38 (20)	8 (4)	30 (16)	*p* = 0.5
	Young adult	87 (46)	17 (9)	70 (37)	*p* = 0.5
	Mature Adult	27 (14)	3 (2)	24 (13)	*p* = 0.4
	Senior	38 (20)	5 (3)	33 (17)	*p* = 0.4
**Breed** **n= 204/204**	European	157 (77)	32 (16)	125 (61)	***p* = 0.03, OR = 3.8 (1–12.9)**
	Other	47 (23)	3 (1)	44 (22)	
**Hair length** **n = 204/204**	Short hair	175 (86)	34 (17)	141(69)	***p* = 0.03, OR = 6.8 (0.8–51.4)**
	Medium/long hair	29 (14)	1 (0.5)	28 (14)	
**Lifestyle** **n = 194/204**	Indoor	139 (72)	21 (11)	118 (61)	*p* = 0.6
	Outdoor	55 (28)	10 (5)	45 (24)	
**Sampling season n = 204/204**	Spring	72 (35)	6 (3)	66 (32)	***p* = 0.01, OR = 0.32 (0.13–0.8)**
	Summer	40 (20)	8 (4)	32 (16)	*p* = 0.6
	Autumn	53 (26)	12 (6)	41 (20)	*p* = 0.2
	Winter	39 (19)	9 (4)	30 (15)	*p* = 0.3
**History of dermatological problems** **n = 192/204**	Yes	38 (16)	11 (6)	27 (14)	***p* = 0.0008, OR = 4.6 (1.8–12)**
	No	154 (84)	10 (5)	144 (75)	
**Unhealthy** **n = 192/204**	Yes	102 (53)	21 (11)	81 (42)	*p* = 0.07
	No	90 (47)	10 (5)	80 (42)	

N = number, OR = odds ratio, CI = confidence intervals.

**Table 3:** Frequency and relationship between symptoms/signs and OE in the population of 204 cats. Significant *p*-values (<0.05) in bold.

Variable n = Number of Subjects for Which Data Were Available	Categories	Total N (%) of Cats	N (%) Cats with Otitis	N (%) Cats Without Otitis	X^2^ *p* Value and OR (95% CI)
**Ear symptoms** **n = 204**	Pruritus	23 (12)	14 (7)	9 (4)	***p* = 0.0000, OR = 39 (12.7–121)**
	Odor	0 (0)	0 (0)	0 (0)	-
	Ear discharge	2 (1)	2 (1)	0 (0)	***p* = 0.0000, OR = 48 (2.2–1027)**
	None	181 (89)	5 (3)	176 (86)	***p* = 0.0000, OR = 0.02 (0.005–0.05)**
**Cerumen amount** **n = 204**	Absent	23 (11)	0 (0)	23 (11)	*p* = 0.09
	Small	101 (50)	1 (0.5)	100 (49)	***p* = 0.0002, OR = 0.01 (0.002–0.16)**
	Moderate	53 (26)	12 (6)	41 (20)	*p* = 0.2
	Abundant	27 (13)	22 (11)	5 (3)	***p* = 0.0000, OR = 55.6 (18–170.7)**
**Ear alterations** **n = 204**	Canal erythema	16 (8)	16 (8)	0 (0)	***p* = 0.0005, OR = 157 (9.2–2686.1)**
	Canal stenosis	7 (3)	7 (3)	0 (0)	***p* = 0.0000, OR = 75.9 (4.2–1361.04)**
	Canal ulceration	1 (0.5)	1 (0.5)	0 (0)	***p* = 0.04, OR = 12.8 (0.5–320.1)**
	Pinna crusts/erosions	7 (3)	6 (2.9)	1 (0.5)	***p* = 0.0065, OR = 19.6 (2.3–166.4)**
	None	183 (89)	20 (10)	163 (80)	***p* < 0.0001, OR = 0.04 (0.01–0.1)**

N = number, OR = odds ratio, CI = confidence intervals.

**Table 4:** Frequency and relationship between abnormal cytology and OE in the population of 204 cats evaluated in this study.

Cytological Parameter	Cats (%) with Otitis N 35	Cats (%) Without Otitis N 169	Fisher’s *p* Value
*Malassezia* spp. ≥12/HPF	10 (28)	1 (0.6)	*p* < 0.000001
Cocci ≥15/HPF	19 (54)	3 (2)	*p* < 0.000001
Rods ≥1/HPF	0 (0)	0 (0)	-
Mites ≥1/HPF	12 (6)	0 (0)	*p* < 0.000001

N = number, HPF = high-power field.

**Table 5:** Mean (±SD) number/HPF of microorganisms found in cats with and without otitis externa.

Microorganisms	Cats with Otitis (+/−DS) N 35	Cats Without Otitis (+/−DS) N 169	*t*-Test *p* Value
*Malassezia* spp./HPF	42 (±29.6)	1.4 (±5.7)	*p* < 0.0001
Cocci/HPF	81.8 (±30.8)	1.3 (±4.7)	*p* < 0.0001
Rods/HPF	0	0	-

N = number, HPF = high-power field.

## Data Availability

The original contributions presented in this study are included in the article. Further inquiries can be directed to the corresponding authors (Roberta Perego and Claudia Avizzano).
